# Cognitive Impairment and Brain Atrophy in Patients with Chronic Kidney Disease

**DOI:** 10.3390/jcm13051401

**Published:** 2024-02-28

**Authors:** Kazuhiko Tsuruya, Hisako Yoshida

**Affiliations:** 1 Department of Nephrology, Nara Medical University, Kashihara 634-8521, Nara, Japan; 2 Department of Medical Statistics, Osaka Metropolitan University Graduate School of Medicine, Osaka 545-8585, Osaka, Japan; hisako.yoshida@omu.ac.jp

**Keywords:** brain atrophy, cognitive impairment, chronic kidney disease, dialysis, exercise

## Abstract

In Japan, the aging of the population is rapidly accelerating, with an increase in patients with chronic kidney disease (CKD) and those undergoing dialysis. As a result, the number of individuals with cognitive impairment (CI) is rising, and addressing this issue has become an urgent problem. A notable feature of dementia in CKD patients is the high frequency of vascular dementia, making its prevention through the management of classical risk factors such as hypertension, diabetes mellitus, dyslipidemia, smoking, etc., associated with atherosclerosis and arteriosclerosis. Other effective measures, including the use of renin–angiotensin system inhibitors, addressing anemia, exercise therapy, and lifestyle improvements, have been reported. The incidence and progression of CI may also be influenced by the type of kidney replacement therapy, with reports suggesting that long-duration dialysis, low-temperature hemodialysis, peritoneal dialysis, and kidney transplantation can have a preferable effect on the preservation of cognitive function. In conclusion, patients with CKD are at a higher risk of developing CI, with brain atrophy being a contributing factor. Despite the identification of various preventive measures, the evidence substantiating their efficacy remains limited across all studies. Future expectations lie in large-scale randomized controlled trials.

## 1. Introduction

In Japan, which is rapidly moving towards a hyper-aged society, the number of patients with cognitive impairment (CI) is increasing rapidly, along with the number of those with chronic kidney disease (CKD) [1,2,3]. In recent years, it has become evident that CKD patients have a higher frequency of CI, and various studies have been conducted to explore the mechanisms, relationships, and strategies concerning CKD and CI [4,5,6,7,8]. Although many findings have been presented, numerous problems remain unresolved [9,10,11]. On the other hand, it has been shown that cerebral atrophy progresses rapidly in patients with CKD, especially in dialysis patients [12,13,14,15,16,17]. Brain atrophy is significantly associated with CI, and atrophy of the frontal lobe and hippocampus is more remarkable in such patients [13,18,19].

This article provides an overview of CI, brain atrophy, and their relationship, and also outlines the factors associated with CI, the mechanisms of CI in CKD, and strategies for addressing CI in CKD.

## 2. Brain Atrophy in Patients with CKD

### 2.1. Brain Atrophy in Patients with Non-Dialysis Dependent CKD (ND)

The brain volume in ND patients has been reported to exhibit more advanced brain atrophy compared to that in healthy controls [20,21,22]. Recently, the relationship between urinary protein and renal function levels and brain volume was examined by magnetic resonance imaging (MRI) in 8630 participants from the general population in Japan, and it was reported that the higher the urinary albumin excretion and the lower the estimated glomerular filtration rate (eGFR), the lower the whole brain volume and the faster the progression of brain atrophy [22]. In contrast, Grasing et al. [23] reported no significant association between brain volumes and the eGFR in a cross-sectional analysis of 1596 participants from the database of the Alzheimer’s Disease Neuroimaging Initiative (ADNI) with a mild to moderately reduced eGFR, as revealed by a multiple linear regression model. While unadjusted associations were initially observed, these were later attributed to the confounding effect of age.

### 2.2. Brain Atrophy in Patients on Hemodialysis (HD)

Brain atrophy has been demonstrated in several reports [12,13,14,15,16,17]. Yoshimitsu et al. [24] evaluated brain atrophy in 55 HD patients and 35 healthy subjects using the ventricular–brain ratio (VBR) quantified from MRI images, and reported that the VBR of HD patients was significantly greater at every 10 years of age in the 30s to 60s age range. In HD patients, the frequency of brain atrophy is high even at a young age, and it is thought that the mechanism cannot be explained by aging alone.

### 2.3. Brain Atrophy in Patients on Peritoneal Dialysis (PD)

In recent years, it has been reported that the brain gray matter volume (GMV) and GMV ratio (GMR) decrease with age, but the white matter volume (WMV) and WMV ratio (WMR) remain unchanged, as revealed through the analysis of brain MRI using statistical parametric mapping (SPM) [25]. Subsequently, we conducted an analysis using SPM on brain MRI data from patients with ND, PD, and HD, comparing both brain volume and the rate of change [26,27].

#### 2.3.1. Comparison between PD and ND Patients

It has been reported that cognitive function is more impaired in PD patients than in ND patients [28]. However, there have been few studies examining the brain volume in PD patients, and none specifically comparing them [29]. Therefore, we conducted a comparison of brain volume and its change over time between the two patient groups [26].

**Figure 1:**
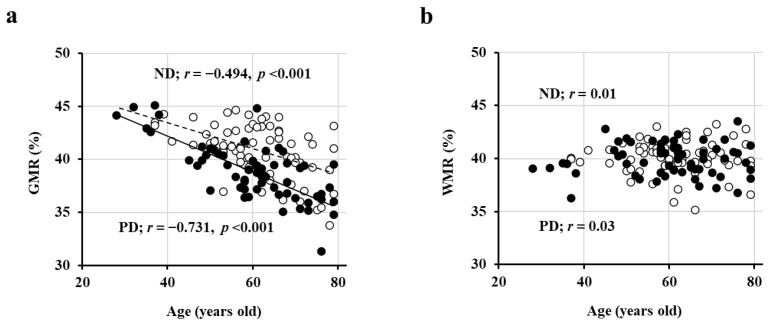
Inverse association of GMR, but not WMR, with age. The association of GMR (**a**), but not WMR (**b**), with age in both PD (closed circles; *n* = 62) and ND (open circles; *n* = 69) patients. Abbreviations: GMR, gray matter volume ratio; ND, non-dialysis-dependent chronic kidney disease; PD, peritoneal dialysis; WMR, white matter volume ratio. Reproduced from Ref. [4].

First, in a cross-sectional study involving 69 patients with ND (ages 61 ± 10 years, 37/32 males and females, eGFR 39 ± 12 mL/min/1.73 m^2^) and 62 PD patients (ages 60 ± 12 years, 41/21 males and females), we observed a significant negative correlation between GMR and age (Figure 1a), whereas WMR did not exhibit a correlation with age (Figure 1b) [26]. Moreover, the regression line was lower in PD patients compared to ND patients (indicating smaller GMR in PD patients at the same age), and this difference further increased with age (Figure 1a) [26].

**Figure 2:**
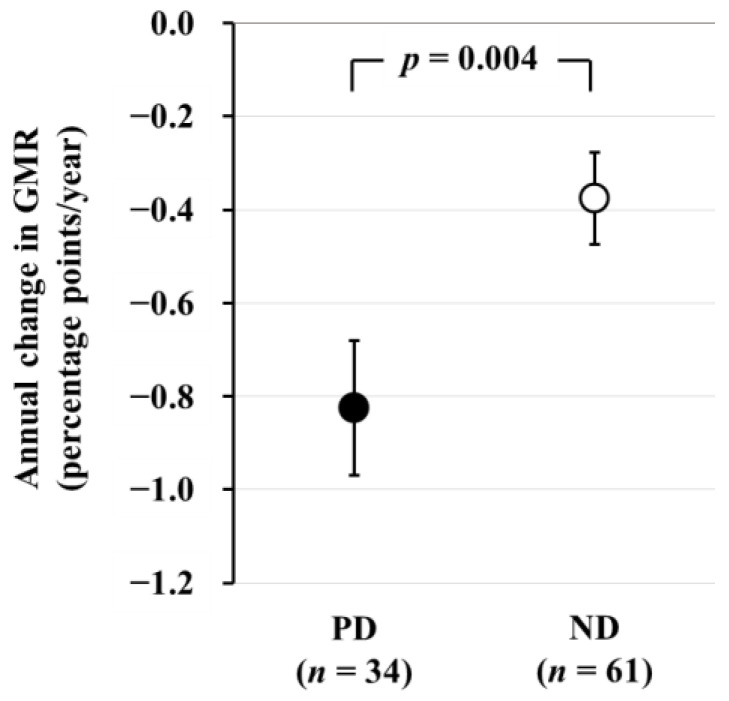
Comparison of the annual change in GMR between PD and ND patients. The annual change in GMR, as determined by subtraction of baseline GMR from GMR after 2 years, is significantly greater in PD patients than in ND patients. Data are least square mean ± standard error. Abbreviations: GMR, gray matter volume ratio; ND, non-dialysis-dependent chronic kidney disease; PD, peritoneal dialysis. Reproduced from Ref. [26].

In a subsequent longitudinal study involving 61 ND patients (age 61 ± 10 years, 32/29 males and females, eGFR 39 ± 12 mL/min/1.73 m^2^) and 34 PD patients (age 60 ± 11 years, 21/13 males and females) who underwent brain MRI examinations after a 2-year interval, we found that the annual change in GMR (AC-GMR) was −0.38 ± 0.10 percentage-points/year in ND patients and −0.83 ± 0.14 percentage points/year in PD patients. This indicates that brain atrophy progressed more than two times faster in PD patients compared to ND patients (Figure 2) [26]. According to a report on general healthy subjects, GMR decreases with age at a rate of 0.2–0.3 percentage points/year, suggesting that brain atrophy progresses three times more rapidly in PD patients than in healthy subjects.

#### 2.3.2. Comparison between PD and HD Patients

**Figure 3:**
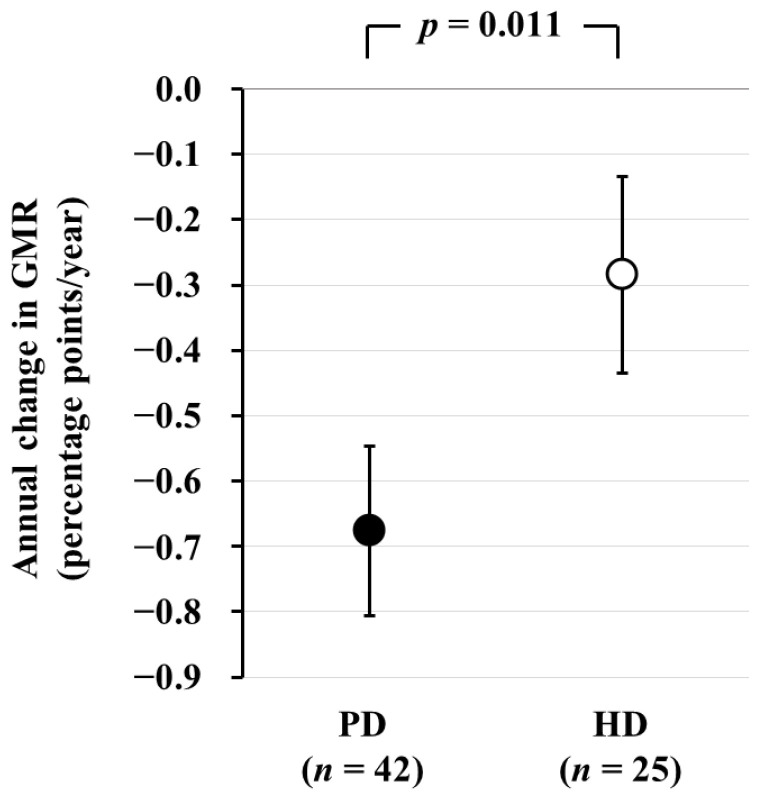
Comparison of the annual change in GMR between PD and HD patients. The annual change in GMR, as determined by subtraction of baseline GMR from GMR after 2 years, is significantly greater in PD patients than in HD patients. Data are least square mean ± standard error. Abbreviations: GMR, gray matter volume ratio; HD, hemodialysis; PD, peritoneal dialysis. Reproduced from Ref. [27].

Next, we conducted a comparison of brain atrophy between PD and HD patients. In total, 73 PD and 34 HD patients who underwent brain MRI were recruited for a cross-sectional analysis. Among them, 42 PD and 25 HD patients who underwent a second brain MRI after 2 years were recruited for a longitudinal analysis. In the cross-sectional analysis, GMR was significantly lower in PD patients than in HD patients [least square mean (LSM): 39.2% vs. 40.0%, *p* = 0.018). AC-GMR was significantly greater in PD patients than in HD patients, and this difference remained statistically significant even after adjusting for potential confounding factors (LSM: −0.68 vs. −0.28 percentage-points/year, *p* = 0.011) (Figure 3) [5]. This study provides evidence of a more accelerated progression of brain atrophy in PD patients compared to HD patients.

Although numerous studies have explored cognitive function in PD and HD patients, our study stands out as the first to directly compare brain volume and the progression of brain atrophy [30,31,32,33,34]. Surprisingly, our findings contradict those of many studies indicating superior cognitive function in PD patients [27].

## 3. Relationship between Brain Atrophy and CI in CKD Patients

The association between brain volume, brain atrophy, and cognitive function has been extensively investigated in studies involving Parkinson’s disease, multiple sclerosis, Alzheimer’s disease, and the general population; yet, few studies have explored the relationship between CI and white matter lesions, as well as brain atrophy, in CKD patients [28,35,36,37,38]. Yeh et al. [39] found a significant correlation between attention and executive function and white matter lesions, but no correlation with brain atrophy.

We conducted a trail making test (TMT) in 95 ND patients who underwent MRI and examined the relationship between TMT scores and brain volume. We demonstrated a significant negative correlation between TMT scores and GMR, and this significance persisted even after adjusting for confounding factors such as age, sex, diabetes mellitus, eGFR, educational history, systolic blood pressure, smoking and alcohol consumption, hemoglobin level, history of cardiovascular disease, and amount of urinary protein (Table 1) [6].

More interestingly, when the brain was partitioned into four regions (frontal lobe, temporal lobe, parietal lobe, and occipital lobe), and the correlation between the GMR and TMT scores in each region was investigated, a significant negative correlation was observed in the frontal and temporal lobes even after adjusting for multivariable variables. No correlation was found in the parietal and occipital lobes. This suggests that the atrophy of the frontal and temporal lobes contributes to the decline in frontal lobe function (executive function), aligning with our initial hypothesis.

## 4. Factors and Pathophysiology Related to CI in Patients with CKD

Several factors contribute to CI in CKD patients, encompassing classical factors such as age, race (black), diabetes, hypertension, and cardiovascular disease, and albuminuria, kidney dysfunction, anemia, oxidative stress, malnutrition and inflammation, and uremic toxins [41]. The types of dementia, in order of frequency, include Alzheimer’s dementia, vascular dementia, and Lewy body dementia. Notably, vascular dementia is more prevalent among patients with CKD.

### 4.1. Albuminuria and Kidney Dysfunction

We investigated the relationship between CKD and the onset of dementia in the Hisayama Study, revealing significant associations between albuminuria and kidney dysfunction and incident dementia. Albuminuria demonstrated associations with both Alzheimer’s dementia and vascular dementia. In contrast, kidney dysfunction, as indicated by an eGFR of <60 mL/min/1.73 m^2^, was specifically associated with vascular dementia but not linked to the development of Alzheimer’s dementia [42].

On the other hand, Viggiano et al.’s review article [43] demonstrates a correlation between mild CI (MCI) and eGFR, with the frequency of MCI increasing as the eGFR decreases. Moreover, when examined by age, the frequency of MCI increases with age in all patients, including the normal control group. However, while the difference in frequency based on the presence of CKD is extremely significant, the variations in frequency among ND, HD, and PD patients are minimal, with almost no difference observed in those under 40 years old. Beyond the age of 40, the distinctions among these patient groups become more pronounced with aging (HD > PD > ND) [43].

Recently, in the Suita study involving 6215 subjects in Japan, it was reported that the odds ratio (95% confidence interval) of MCI for the Mini-Mental State Examination (MMSE) score < 26 was significantly higher, at 1.49 (1.22–1.83) and 2.35 (1.69–3.26) for participants with an eGFR of 45–59.9 mL/min/1.73 m^2^ and <45 mL/min/1.73 m^2^, respectively, compared to those with eGFR > 60 mL/min/1.73 m^2^ [44].

### 4.2. Sleep Duration and Sleep Quality

Recent reports [45,46,47,48] have indicated an association between sleep duration, sleep quality, and CI in patients with CKD. Among ND patients, CI is linked to sleep apnea syndrome and sleeping longer than 9 h [45,46]. In PD patients, narcolepsy is correlated with lower modified MMSE scores [47]. Furthermore, an analysis of 2286 cases in the United States Renal Data System (USRDS) data revealed an association between sleep disturbances and Kidney Disease Quality of Life Cognitive Function (KDQOL-CF) scores in HD patients [48].

### 4.3. Frailty

Recently, the association between frailty and CI has been documented in ND, HD, PD patients and kidney transplant recipients [49,50,51,52,53,54,55,56]. It has been suggested that the mechanisms underlining this relationship involve an elevated concentration of Aβ in the cerebrospinal fluid and increased Aβ deposition in the brain, which are attributed to reduced physical activity. Liang et al. [57] also reported that among 69 older adults with normal cognitive function, those who were physically active and met or exceeded the exercise recommendations of the American Heart Association exhibited significantly lower levels of Aβ deposition, as measured with positron emission tomography, and higher levels of Aβ42 in the cerebrospinal fluid compared to inactive individuals who did not meet the recommendation. Alternatively, other animal studies have demonstrated that physical training enhances angiogenesis, synaptogenesis and neurogenesis, particularly in the hippocampus and gyrus dentatus, and initiates the upregulation of numerous neurotrophic factors in the brain [58,59], especially in the hippocampus [60,61].

An elevation of BDNF concentrations is linked to an increase in hippocampal size and an enhancement of spatial memory and learning performance [62]. Insulin-like growth factor-1 (IGF-1) has been demonstrated to boost BDNF signaling in response to activity stimulation. The neurogenesis induced by exercise in the rat hippocampus is inhibited by the injection of a serum that blocks IGF-1 from leaving the bloodstream and entering the cerebrospinal fluid [63]. IGF-1 also contributes greatly to the exercise-induced effects of BDNF on recall [64]. The neuronal uptake of IGF-1 is stimulated by exercise, and these neurons then show signs of activity and increase their expression of BDNF [65].

## 5. Mechanisms of CI in CKD

### 5.1. Atherosclerosis and Cerebrovascular Disease

Cerebrovascular disease in CKD primarily arises from a synergistic interplay between classical and non-classical mechanisms. The classical risk factors and mechanisms encompass hypertension, diabetes, atrial fibrillation, carotid artery disease, heart failure, obesity, and dyslipidemia, which are all frequently comorbid in CKD. Non-classical risk factors, including chronic inflammation, uremic toxins, reactive oxygen radicals, anemia, and bone mineral disorders, are believed to contribute to the risk of cerebrovascular accidents and CI by inducing vascular injury and endothelial dysfunction [66]. Uremia is considered to promote atherosclerosis through protein carbamylation and contribute to dyslipidemia.

Tasmoc et al. [67] investigated the association between pulse wave velocity (PWV), reflecting arterial stiffness, and cognitive function (TMT, MMSE, etc.) in a study involving 72 HD patients. They reported a significant correlation between elevated PWV values and CI. Additionally, in the COPE study [68], a prospective multicenter cohort study in the Netherlands involving 85 elderly CKD patients including ND and dialysis patients, the relationship between PWV measured by MRI and cognitive function was examined. The study found significant correlations between PWV and all aspects of memory, executive function, and psychomotor speed. In particular, in executive function, a significant association persisted even after adjusting for age, gender, and education.

On the other hand, in the PACE study of 330 dialysis patients [69], it was reported that the augmentation index (AI) and central pulse pressure (cPP), which more notably reflect systemic arterial stiffness than PWV, were significantly associated with CI compared to PWV. Recently, Nishimura et al. [70] reported in a study of 100 HD patients (mean age 67.9 years old, mean history of dialysis 7.3 years) that those with an ankle brachial index (ABI) ≥ 1.06 (*n* = 69) had significantly higher Montreal Cognitive Assessment (MoCA) scores (25.5 ± 3.9 vs. 22.3 ± 4.6) compared to patients with an ABI < 1.06 (*n* = 31). Moreover, the ABI and MoCA showed a significant positive correlation in the multiple regression analysis.

These studies suggest that arterial stiffness may be associated with CI and could represent the underlying mechanism of incident CI in elderly CKD patients.

### 5.2. Hypotension and Decrease of Regional Cerebral Blood Flow during HD

In HD patients, there are specific factors related to HD that influence the risk of cerebrovascular accidents, including cerebral hypoperfusion, enhanced arteriosclerosis, and blood pressure fluctuations [71]. The mean flow velocity of cerebral arteries has been shown to decrease significantly during HD, leading to transient cerebral ischemia and ischemic white matter lesions over time [72].

One of the factors associated with CI in HD is a rapid decrease in blood pressure during HD. Mizumasa et al. [73] conducted a three-year longitudinal study examining the association of rapid hypotension during HD with brain ischemia and brain atrophy using brain MRI. The study revealed a positive association between the total number of rapid hypotension episodes during the HD sessions and the number of lacunar infarctions, along with the degree of frontal lobe atrophy over the three-year period.

In addition, the relationship between decreased regional cerebral blood flow (rCBF) and CI has been reported. Kobayashi et al. [74] examined the cognitive function of rCBF and MMSE measured by SPECT in 54 HD patients, and reported that the rCBF in the middle cerebral artery perfusion region was significantly lower than in other areas in patients with reduced MMSE.

It has also been reported that cerebral oxygen saturation (rSO_2_) decreases during HD, as observed using near-infrared spectroscopy (NIRS) [75]. Malik et al. [76] reported that rSO_2_ decreased most significantly at 35 min after the initiation of HD. More recently, MacEwen et al. [72] highlighted that the decrease in rSO_2_ observed was strongly influenced by the reduction in blood pressure during HD. They found that a decrease in mean blood pressure by 10 mmHg during HD increased the risk of cerebral ischemia (rSO_2_ decreased by 15% or more) by 3%, and the risk rose rapidly when the mean blood pressure dropped below 60 mmHg.

Recently, there has been extensive research on the relationship between cerebral ischemia and CI in dialysis patients. The extent of reduction in the mean cerebral blood flow velocity during HD correlates with the degree of cognitive decline and the deterioration of white matter lesions after 12 months. Additionally, the rSO2 of the left frontal lobe, measured by NIRS, is significantly correlated with the MoCA score. Furthermore, a correlation has been reported between the mixed venous oxygen saturation and MoCA score in the left internal cerebral vein [71,77,78].

### 5.3. Oxidative Stress

**Figure 4:**
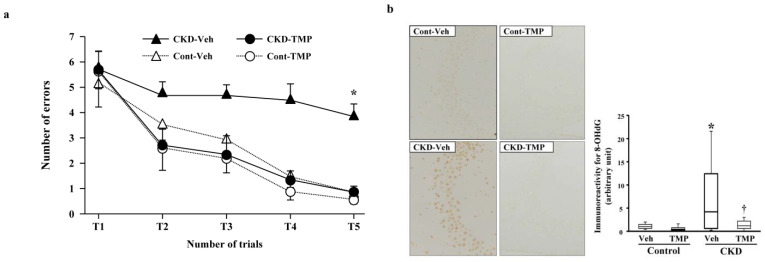
Effect of tempol on prevention of uremia-induced spatial working memory dysfunction and inhibition of 8-OHdG accumulation in the hippocampal CA3 region. (**a**) The numbers of errors during radial arm water maze test on the fifth day in Cont-Veh (white triangle), CKD-Veh (black triangle), Cont-TMP (white circle) and CKD-TMP (black circle) mice are shown. The number of errors in CKD-TMP mice is significantly decreased to levels similar to those observed in control mice, and significantly lower than CKD-Veh mice. The ends of the box represent the upper and lower quartiles; thus, the box spans the interquartile range. The median is marked by a vertical line inside the box. The two lines outside the box that extend to the highest and lowest observations represent the whiskers. * *p* < 0.05 versus the other three groups. (**b**) Effect of TMP on prevention of oxidative DNA damage generation. Representative microphotographs of 8-OHdG immunostaining in the hippocampal CA3 region from each group are shown. Magnification: ×200. Quantitative analysis of 8-OHdG-positive neurons in the hippocampal CA3 region is shown. 8-OHdG immunoreactivity in the hippocampal CA3 region is significantly higher in CKD-Veh mice than TMP-treated CKD mice. The ends of the box represent the upper and lower quartiles; thus, the box spans the interquartile range. The median is marked by a vertical line inside the box. The two lines outside the box that extend to the highest and lowest observations represent the whiskers. * *p* < 0.05 versus Cont-Veh mice. † *p* < 0.05 versus CKD-Veh mice. Abbreviations: CA3, cornu ammonis 3; CKD, chronic kidney disease; Cont, control; 8-OHdG, 8-hydroxy-2′-deoxyguanosine; TMP, tempol; Veh, vehicle. Reproduced from Ref. [79].

In order to elucidate the mechanism of CI in CKD, we conducted the following experiments using CKD mice created through 5/6 nephrectomy. At 8 weeks post-modeling, we performed the water maze test and evaluated the learning function of CKD mice compared to sham-operated control mice. Subsequently, we conducted pathological and immunohistological examinations using the extracted brains. In the hippocampus of CKD mice, degenerated cells with nuclear condensation (pyknotic cells) appeared along with the accumulation of 8-hydroxy-2′-deoxyguanosine (8-OHdG). The learning ability of CKD mice was significantly decreased in the water maze test. Conversely, in CKD mice administered with an antioxidant (Tempol), the accumulation of 8-OHdG and pyknotic cells in the hippocampus was minimal, and the results of the water maze test were equivalent to those of the control mice (Figure 4) [79]. These findings suggest that oxidative stress associated with CKD plays a significant role in neuronal damage in the brain and the decline of learning ability in CI in CKD.

### 5.4. Insoluble Tau Protein

Recently, Matsuki et al. [80] demonstrated CI in CKD mice. The researchers extracted the hippocampus from both CKD and healthy mice, performing a proteomic analysis by partitioning the hippocampus into soluble and insoluble fractions through salting and salting out. The results indicated increased levels of insoluble tau protein and RNA splicing-related proteins in the brains of CKD mice, akin to findings in Alzheimer’s disease. The study further revealed elevated levels of insoluble phosphorylated tau protein in the hippocampus and cerebral cortex of CKD mice, along with increased immunoglobulin heavy chains. This suggests that the dysfunction of the blood–brain barrier (BBB) enhances substance permeability. Additionally, a multivariable logistic regression analysis of 980 CKD patients, considering CI as the objective variable, identified that elevated blood urea nitrogen and a low nutritional status are strong risk factors for dementia. This implies that the accumulation of urea and other uremic substances is secondarily associated with CI rather than the pure kidney filtration function itself.

## 6. Measures for CI in Patients with CKD

Interventions aimed at reducing the incidence of CI in patients with CKD include the management of classical cardiovascular risk factors, using renin–angiotensin system (RAS) inhibitors, maintaining strict blood pressure management, incorporating cognitive training, engaging in regular exercise, and more [81].

### 6.1. RAS Inhibitors

When the angiotensin II receptor antagonist telmisartan was administered to mice in a CKD model through 5/6 nephrectomy, both the accumulation of 8-OHdG in the brain hippocampus and the decline in learning capacity were suppressed, similar to the effects observed with Tempol administration [82]. Clinically, a meta-analysis has demonstrated the inhibitory effect of RAS inhibitors on Alzheimer’s disease and age-related cognitive decline [83].

### 6.2. Strict Antihypertensive Management

It has been suggested that strict antihypertensive management may suppress the onset of dementia. In the SPRINT-MIND Study [84], a randomized controlled trial (RCT) comparing the incidence of CI between the intensive management group, with a target systolic blood pressure of less than 120 mmHg, and the standard control group with a target of less than 140 mmHg, among hypertensive patients aged 50 years and older with no history of diabetes or stroke, the incidence rate of suspected dementia, set as a primary endpoint, was 7.2 and 8.6 cases per 1000 person years in the intensive management group and in the standard management group, respectively [hazard ratio (HR) 0.83, 95% confidence interval 0.67–1.04, *p* = 0.10]. Although the difference did not reach statistical significance, there was a trend towards a lower incidence in the intensive management group. Furthermore, the study demonstrated that the risks of MCI (HR 0.81, 95% confidence interval 0.69–0.9, *p* = 0.007) and the composite outcome of MCI and suspected dementia (HR 0.85, 95% confidence interval: 0.74–0.97, *p* = 0.01) as secondary endpoints were significantly lower in the intensive management group.

### 6.3. Management of Anemia

It has been revealed that the treatment of anemia with recombinant human erythropoietin (rHuEPO) improves brain function. In a study by Grimm et al. [85] involving 15 chronic HD patients, the event-related potential P300 of brain waves was measured. They reported a significant shortening of the peak latency of P300 following an improvement in anemia with rHuEPO (hematocrit 22.7% → 30.6%), indicating an observed enhancement in higher cognitive functions. Additionally, Singh et al. [86] reported similar effects in both ND and dialysis patients with CKD.

### 6.4. Exercise Therapy

In recent years, concerns regarding frailty have emerged among elderly dialysis patients. This condition is characterized by a decline in skeletal muscle mass and strength, the onset of sarcopenia, and a decline in overall physical and cognitive functions associated with aging. This frailty contributes to compromised daily functioning and the manifestation of physical and mental vulnerabilities. Consequently, the importance of exercise therapy in addressing these challenges has gained recognition. Exercise therapy has been reported not only to enhance physical function but also to contribute to improvements in cognitive function. Manfredini et al. [87] conducted a study involving 296 dialysis patients, randomly assigning them to a group performing 10 min of walking exercise three times a week at home (exercise group) or a control group without exercise. After six months, they evaluated the 6 min walking test, sit-to-stand test, and the KDQOL-SF scale to measure the quality of life (QOL) of kidney disease patients. The results indicated a significant extension in walking distance and a noteworthy reduction in the sit-to-stand time in the exercise group compared to the control group. Additionally, the cognitive function score in the KDQOL-SF was significantly improved in the exercise group. Furthermore, in a recent study, Otobe et al. [88] investigated 53 outpatient cases at CKD stages G3–G4. The patients were randomly assigned to an exercise group (*n* = 27), where group exercise training occurred once a week at a facility, coupled with self-directed exercise at home at least twice a week for 24 weeks, and a control group (*n* = 26). The study aimed to compare changes in cognitive function between the two groups. The results demonstrated that, in comparison to the control group, the exercise group exhibited significant improvements in both delayed and immediate recall scores on the Wechsler Memory Scale–Revised Logical Memory.

In an RCT by McAdams-DeMarco et al. [89], not only physical exercise during HD using an ergometer but also cognitive training (brain training games using tablets) demonstrated an improvement in cognitive function. The study reported a significant reduction in the TMT time, indicating a positive effect on cognitive enhancement. Recently, Liu et al. [90] conducted a meta-analysis of these RCTs. The findings indicated that exercise during or between HD sessions significantly improved cognitive function in HD patients. Notably, the effects were particularly significant when the duration was 30 min or more, performed at least three times a week, and continued for a minimum of 16 weeks.

### 6.5. Interventions Targeting Multiple Factors such as Lifestyle Improvement

Dementia is a multifactorial condition influenced by various factors, and it is considered crucial to simultaneously address interventions targeting multiple factors. In the FINGER study conducted in Finland [91], individuals at risk of developing dementia, such as those with MCI, were randomly assigned to a lifestyle improvement group receiving interventions in exercise, diet, cognitive training, and vascular risk management, or a control group. The study aimed to investigate changes in cognitive function. As a result, cognitive function significantly improved in the lifestyle improvement group, showing a remarkable 25% enhancement compared to the control group. Moreover, significant improvements were observed in the participants’ executive function and processing speed, with 83% and 150% improvements in executive function and processing speed, respectively. A trend toward improvement was also noted in memory function. Given the historical difficulty of preventing dementia for individuals at high risk, this study has made a significant impact on dementia researchers. At the Alzheimer’s Disease International Conference in 2019, ten risk factors for dementia (ApoE ε4, education, hearing loss, hypertension, obesity, smoking, depression, diabetes, reduced physical activity, social isolation) and nine protective factors (excluding ApoE ε4) were revealed to play a role in prevention [92].

### 6.6. Extension of Dialysis Session Time

It has been suggested that extended HD sessions may contribute to an improvement in cognitive function. Ok et al. [93] conducted a prospective, case–control study involving 247 patients who consented to 8 h HD sessions three times a week and a control group that consisted of 247 patients receiving 4 h HD sessions three times a week, matched for age, gender, diabetes mellitus, and dialysis history over a 12-month period. The study compared prognosis, cognitive function, QOL, etc., revealing a significant improvement in memory function in the long-hour HD group. Conversely, Chertow et al. [94] reported in the FHN Trial that frequent HD six times a week did not result in improved cognitive function.

### 6.7. Decrease in Dialysate Temperature

Eldehni et al. [95] reported in an RCT that reducing the dialysate temperature by 0.5 °C can inhibit the progression of cerebral white matter lesions over one year, and a meta-analysis has also shown that low-temperature HD suppresses the risk of lowering blood pressure during HD [96]. Currently, an RCT is being conducted to investigate the effect of suppressing the decline in cognitive function by lowering the dialysate temperature [97]. Dialysis practices including the session frequency, duration and dialysate temperature vary significantly between centers, which could obscure the potential benefits of optimizing prescriptions. Standardizing dialysis protocols in future comparative effectiveness trials is necessary.

### 6.8. Prevention and Measures against Lowering Blood Pressure and Cerebral Ischemia during and after HD

To prevent a drop in blood pressure during HD, it is crucial to set dry weights appropriately and minimize the rate of fluid removal per hour. Diabetic patients, in particular, exhibit severe systemic arteriosclerosis, along with stenotic and occlusive lesions in the intra- and extracranial main arteries. They are more susceptible to hypotension during HD and orthostatic hypotension after HD. In such cases, fluid and blood pressure management becomes exceptionally important due to the impaired autoregulation of the brain, increasing the risk of hemodynamic cerebrovascular accidents [98]. Nevertheless, vasopressor administration may be effective in cases where blood pressure decreases during dialysis. Fujisaki et al. [99] reported that the reduction in cerebral blood flow resulting from orthostatic hypotension after HD can be prevented by the administration of vasopressor drugs such as midodrine hydrochloride and droxidopa. Notably, droxidopa exhibits a remarkable effect in retaining cerebral blood flow. The mechanism involves the dilation of cerebral blood vessels by activating adrenergic receptors, particularly β receptors that cross the BBB, in addition to inducing peripheral vasoconstriction. It is speculated that this mechanism contributes to the preservation of cerebral blood flow.

### 6.9. PD

It has been reported that cognitive function is better preserved in PD compared to HD patients. According to the statistical survey conducted by the Japanese Society for Dialysis Therapy at the end of 2018, the frequency of dementia was 12.68% in HD patients and 5.62% in PD patients, indicating a lower occurrence of dementia in PD than HD patients [30]. While this difference is believed to be primarily due to biases in the choice of treatment, observational studies adjusted for patients’ backgrounds [31,32] and meta-analyses [33,34] have reported a lower risk of CI in PD compared to HD. Thus, the possibility that this factor contributes to the observed difference cannot be ruled out.

### 6.10. Kidney Transplantation (KTx)

Cognitive function has been reported to improve with KTx; Chhabra et al. [100] conducted a study measuring P300 in 20 KTx recipients aged 18–50 years before and 3 months after KTx. They demonstrated that P300 latency, which was prolonged before KTx, shortened to a level comparable to healthy subjects after KTx, and reported that cognitive function improved with an improvement in anemia. On the other hand, Joshee et al. [101] compared cognitive function between pre- and post-KTx, KTx and non-KTx patients, and KTx recipients and healthy subjects. They reported an improvement in cognitive function after KTx but noted that it did not reach the level observed in healthy subjects.

### 6.11. Valerian

As an agonist of the adenosine A1 receptor, valerian inhibits cholinergic transmission, enhances the frequency intensity of delta, theta, and alpha waves in the frontal cortex, and exerts a sedative effect. Samaei et al. [102] examined the impact of valerian on cognitive function in a double-blind crossover RCT and reported an improvement in MMSE scores. However, no changes were observed in brain wave patterns.

### 6.12. Melatonin

More recently, a study involving 102 HD patients examined the impact of melatonin administration on sleep quality (PSQI) and changes in the MoCA were investigated [103]. The patients were divided into a melatonin group, receiving melatonin, and a control group without melatonin. Significant improvements in both the PSQI and MoCA scores were observed in the melatonin group, whereas no significant differences were noted in the control group before and after the study period [103].

## 7. Discussion

Numerous challenges lie ahead with regard to CKD and CI, including elucidating their mechanisms, establishing preventive and therapeutic approaches, creating guidelines for medical practices related to environmental considerations for dementia in dialysis patients, and developing legal frameworks for withholding, withdrawing or discontinuing dialysis. In this rapidly aging society, the issues of CKD and CI are expected to become increasingly significant. Early preventive efforts are deemed crucial, and a thorough understanding of the interrelationship between CKD and CI is essential to implement appropriate measures.

The explanation for the discrepancy between the findings in our study, where brain atrophy progressed more rapidly in PD patients than in HD patients, and the findings in previous reports indicating superior cognitive function in PD patients compared to HD patients is not clear. However, possible explanations include the following: All of the aforementioned reports on CI were observational studies, not RCTs. Selection bias in determining dialysis modalities may have therefore influenced the results. Alternatively, our findings were based on a small sample size and are not conclusive. Therefore, further extensive research, including studies of brain volume and cognitive testing, is required to address this discrepancy. While RCTs are ideal, it is impractical to randomly assign dialysis modalities. Thus, prospective observational studies with a larger sample size and statistical adjustment for treatment selection bias are needed to resolve this discrepancy.

There are a lot of limitations in our manuscript. First, many of the discussed studies have relatively small sample sizes, with some having fewer than 100 participants. Small samples increase the risk of sampling bias and reduce generalizability. Larger, multicenter studies with sample sizes in the thousands are needed to generate more precise effect estimates and ensure that the findings apply to wider CKD populations. Second, there is a lack of high-quality RCTs evaluating treatments for cognitive impairment. Observational designs allow only associative conclusions. Large, placebo-controlled RCTs are needed to definitively establish the causal impacts of proposed interventions like exercise, cognitive training, etc., on outcomes. Third, the complex medical histories of CKD patients make isolating the effect of CKD itself difficult. Many patients have diabetes, hypertension or cardiovascular disease, which all impact cognition. Future studies must appropriately account for these key confounders in design and analysis. Fourth, studies use heterogeneous assessment tools ranging from TMT to the MoCA to diagnose CI. Varied criteria and cutoffs reduce the comparability of prevalence and treatment effects between studies. More research is needed to standardize diagnostic tests and thresholds for CI in CKD patients. Fifth, evidence comes predominantly from cross-sectional studies, limiting causal inference about how CKD contributes to cognitive decline over time. The collection of longitudinal data with repeated neurocognitive testing is essential to establish the temporal relationships between risk factors, brain changes, and cognition. Sixth, many studies fail to consider how differences in age, sex and education status may confound the observed associations between kidney function and cognition. Adjusting for sociodemographic variables as covariates is important to accurately characterize the effects of CKD. Seventh, few studies analyze the sustainability of interventions for CI beyond six months. Assessing longer-term impacts up to 1–2 years is important to determine the durability of early benefits and guide ongoing care. Eighth, simultaneously targeting multiple risk factors may confer greater benefits, but few studies take such an approach. High-quality, large pragmatic trials randomizing participants to multimodal interventions (e.g., exercise + diet + cognitive training) versus standard care are needed.

Continuous research efforts are anticipated to accumulate both foundational and clinical evidence, leading to the establishment of feasible and effective preventive and therapeutic strategies in real-world clinical settings.

## 8. Conclusions

In conclusion, patients with CKD are at a higher risk of developing CI, with brain atrophy being a contributing factor. Despite the identification of various preventive measures, the evidence substantiating their efficacy remains limited across all studies. Future expectations lie in large-scale RCTs.

## Figures and Tables

**Table 1:** Univariable and multivariable-adjusted regression analyses of correlation between whole-brain GMR and TMT scores in all participants.

		TMT-A	TMT-B	ΔTMT
Univariable analysis	Standardized β	−0.490	−0.516	−0.476
	*p*	<0.001	<0.001	<0.001
Model I	Standardized β	−0.442	−0.467	−0.432
	*p*	<0.001	<0.001	<0.001
Model II	Standardized β	−0.394	−0.423	−0.393
	*p*	0.002	<0.001	0.003
Model III	Standardized β	−0.349	−0.362	−0.332
	*p*	0.012	0.006	0.013

Model I: Multivariable analysis adjusted for sex and age. Model II: Model I + diabetes mellitus, estimated glomerular filtration rate, and education. Model III: Model II + systolic blood pressure, smoking habits, drinking habits, hemoglobin, previous history of cardiovascular disease, and log-transformed urinary protein to creatinine ratio. Abbreviations: GMR, gray matter volume ratio; TMT, trail making test. Reproduced from Ref. [40].

## Data Availability

Not applicable.
